# Migration and health: moving towards a diversity-oriented public health monitoring at the Robert Koch Institute

**DOI:** 10.25646/11141

**Published:** 2023-03-21

**Authors:** Claudia Hövener, Lothar H. Wieler

**Affiliations:** 1 Robert Koch Institute, Berlin Department of Epidemiology and Health Monitoring; 2 Robert Koch Institute, Berlin Institute Management

## Abstract

Summarizing categories, such as migration background or history of migration, do not reflect the diversity and heterogeneity of the population living in Germany and their health.

A differentiated description of the health situation of people with a history of migration should consider migration-related, social, and structural determinants of health as well as their interactions.

The findings obtained in the ‘Improving Health Monitoring in Migrant Populations (IMIRA)’ projects will help to improve the inclusion of people with a history of migration in future studies as well as in the RKI panel. This will enable an adequate description of the health situation of people with a history of migration and therefore of the general population in Germany.

In future studies, the health status of people who have not been well included in health surveys so far, such as people who are not listed at the registration office, should be monitored. For this purpose, continuous development of sampling and survey methods is necessary.

Germany is a country of immigration and has a long history of different migration movements, such as labour migration since the 1950’s or according to the European Union (EU) Freedom of Movement Act, and refugee migration in the context of wars and political conflicts. People with a history of migration represent a large proportion of the population living in Germany. In 2021, 27% were attributed a statistically defined migration background. Moreover, 17% of all people living in Germany were migrants themselves, and 13% had a citizenship other than German [2].

One’s own or a familial history of migration alone does not make one healthier or sicker. However, there are various factors before, during, and after migration that can affect one’s health status [3]. People with a history of migration differ in terms of their participation opportunities, socio-economic situation, German language proficiency, as well as motives and circumstances of their own or their familial migration process. This heterogeneity also goes hand in hand with different health opportunities, risks, and healthcare needs and should be reflected in data analysis and public health reporting on migration and health. Only by considering this diversity, is it possible to make differentiated statements about the health status of people with a history of migration. Thus, it is necessary not only to compare broadly categorised groups in analyses of public health data, such as people with or without a migration background, but also to consider aspects such as socio-economic situation, duration of residence, reasons for migration, German language proficiency, or experiences of discrimination in a differentiated manner [4].

Since 2016, the Robert Koch Institute (RKI) has been working on the IMIRA (Improving Health Monitoring in Migration Populations) and IMIRA II projects to expand public health monitoring in a diversity-oriented way. This entails the improved inclusion of people with a history of migration in studies in order to describe their health status in a more differentiated way within the context of public health reporting. Within IMIRA (2016–2019), feasibility studies were conducted to evaluate how to improve the inclusion of people with a history of migration in health surveys [5]. New concepts [6] and core indicators [7] were developed to describe their health situation, and relevant (secondary) data sources were explored for analysis.

The main objective of the IMIRA II project (2019–2023) is to describe the current health status of people with a history of migration in a differentiated manner. Findings on the improved inclusion in health surveys obtained in IMIRA were implemented here, and the health interview survey GEDA Fokus was conducted among people with five selected citizenships [8]. For GEDA Fokus, a register-based sample was drawn, and more than 6,000 people with Croatian, Italian, Polish, Syrian, or Turkish citizenship were interviewed about their health situation. Different multilingual survey modes (online, paper-based, face-to-face, telephone) and materials were offered, as well as questionnaires on the health situation, social and structural determinants of health, and direct and indirect effects of the COVID-19 pandemic.

The first results of GEDA Fokus are presented in the two original articles in this issue. In the article ‘Health of people with selected citizenships: results of the study GEDA Fokus’, some of the core indicators identified in IMIRA, such as self-assessed health or the prevalence of depressive symptoms, are analysed according to various migration-related and social determinants of health. The results show associations between the sense of belonging to the society in Germany and self-reported experiences of discrimination in the healthcare sector and various health outcomes. The article ‘COVID-19 vaccination status among people with selected citizenships: results of the study GEDA Fokus’ shows an educational and age gradient associated with the utilisation of COVID-19 vaccination. Both articles demonstrate that, depending on the health outcome, different migration-related, social, and structural determinants are relevant for explaining differences in health outcomes. The results thus underline that a differentiated, more in-depth consideration of various influencing factors is essential.

The article ‘Recommendations for collecting and analysing migration-related determinants in public health research’ addresses this issue. In addition to the recommendation of a minimum set of indicators to describe the migration status, suggestions are made for further relevant explanatory factors that should be considered in future data collection and analysis. Additionally, the article recommends refraining from using broad summarising categories, such as migration background. Instead, it encourages applying individual indicators that are relevant to answering the research question at hand. Therefore, besides considering one’s own or familial migration experience, it is essential to focus on specific migration-related determinants as well as on social and structural factors affecting health.

We hope that the results presented in this issue can be of use for further advancements towards a diversity-oriented public health research. Thank you for your interest.
